# A Model of Trust in Online COVID-19 Information and Advice: Cross-Sectional Questionnaire Study

**DOI:** 10.2196/59317

**Published:** 2025-02-13

**Authors:** Elizabeth Sillence, Dawn Branley-Bell, Mark Moss, Pam Briggs

**Affiliations:** 1 Department of Psychology Northumbria University Newcastle upon Tyne United Kingdom

**Keywords:** eHealth, electronic health, digital intervention, trust, online information seeking, scientific credibility, digital resources, COVID-19, SARS-CoV-2, respiratory, infectious, pulmonary, pandemic, public health, health information, global health, surveys, social media

## Abstract

**Background:**

During the COVID-19 pandemic, many people sought information from websites and social media. Understanding the extent to which these sources were trusted is important in relation to health communication.

**Objective:**

This study aims to identify the key factors influencing UK citizens’ trust and intention to act on advice about COVID-19 found via digital resources and to test whether an existing model of trust in eHealth provided a good fit for COVID-19–related information seeking online. We also wished to identify any differences between the evaluation of general information and information relating specifically to COVID-19 vaccines.

**Methods:**

In total, 525 people completed an online survey in January 2022 encompassing a general web trust questionnaire, measures of information corroboration, coping perceptions, and intention to act. Data were analyzed using principal component analysis and structural equation modeling. The evaluation responses of general information and COVID-19 vaccine information were also compared.

**Results:**

The principal component analysis revealed 5 trust factors: (1) credibility and impartiality, (2) familiarity, (3) privacy, (4) usability, and (5) personal experiences. In the final structural equation modeling model, trust had a significant direct effect on intention to act (β=.65; *P*<.001). Of the trust factors, credibility and impartiality had a significant positive direct effect on trust (β=.82; *P*<.001). People searching for vaccination information felt less at risk, less anxious, and more optimistic after reading the information. We noted that most people sought information from “official” sources. Finally, in the context of COVID-19, “credibility and impartiality” remain a key predictor of trust in eHealth resources, but in comparison with previous models of trust in online health information, checking and corroborating information did not form a significant part of trust evaluations.

**Conclusions:**

In times of uncertainty, when faced with a global emergent health concern, people place their trust in familiar websites and rely on the perceived credibility and impartiality of those digital sources above other trust factors.

## Introduction

### Background

The COVID-19 pandemic understandably led to an increase in “official” sources of information and advice from politicians, public health officials, clinicians, and scientists. This public-facing information was communicated via the mainstream press, through live-streamed press briefings, and online. However, “unofficial” sources of information were also circulated, primarily via social media. For individuals, access to good quality information during the pandemic was critical, not least because official messaging was constantly being updated in relation to recommended or mandated behaviors such as social distancing, mask-wearing, and self-isolation.

During this time, many people sought their information online [1] through websites, social media, and mobile apps. People looked for information on the signs and symptoms of the virus, measures to avoid catching and spreading the virus, self-care once infected, and vaccination information. In addition to health advice, people also sought related information on rules and guidance regarding self-isolating, masks, and social distancing.

Accurate and appropriate health communication is an important tool in tackling any pandemic and it can directly influence individuals’ affective and behavioral responses to a crisis [2]. In relation to the COVID-19 pandemic, studies have shown that access to a larger and more diverse set of information sources led to increased worry [1,3] and greater confusion, in part because of the infodemic of misinformation and rumors that were promoted about the pandemic [4]. The UK Government’s approach to tackling COVID-19 relied upon broad public trust, but issues with inconsistent and unclear messaging, as well as general political mistrust, were apparent [5]. In short, it sometimes became difficult for people to know who to trust in relation to taking appropriate actions to reduce the spread of COVID-19 and minimize personal risk.

Against this backdrop, the aim of this study was to understand more about the digital resources people in the United Kingdom used for COVID-19–related information and the extent to which they trusted these resources. Although we know that online health formed a key source of information for many people during the pandemic, we do not know how people evaluated these digital sources and what factors were important in trusting the information, the source, and ultimately deciding whether or not to act on the advice given. We also wished to test whether an existing model of trust in eHealth provided a good fit for COVID-19–related information seeking online. We begin by briefly reviewing the literature on trust and eHealth before introducing the COVID-19 context and outlining the study objectives.

### Trust in Online Health Information

Over the last 20 years, research has consistently pointed to the importance of both the design and the content of websites in terms of establishing trustworthiness [6,7]. Commonly reported indicators of trust and credibility include site owners or sponsors; consensus among multiple sources; characteristics of writing and language; advertisements; content authorship; and interface design [8]. Related studies have looked at the quality of web-based health information and have highlighted navigability, aesthetics, and ease of understanding as important factors [9]. As digital resources for health have developed and diversified, we have seen a move away from government and medically driven sources towards more charity and patient-led sites [10] and the use of social media [11,12] meaning that shared patient experience has also become a critical factor in determining trust and appropriateness of online advice [13].

Despite concerns about the quality and reliability of some digital sources [14], they are often well-used and well-liked. Interestingly, they are not necessarily trusted and the advice they contain is not always acted upon. In part, this may relate to a dislike in the United Kingdom for commercial funding models underpinning health websites [10]. A recent model of trust in eHealth [15] found that credibility and impartiality are the key predictors of trust in eHealth websites and noted that websites containing patients’ experiences can have a positive impact on trust but only if those sources have been checked against other sources first. The authors also noted that the need to corroborate digital information sources may be reduced in cases where there is strong familiarity with a well-used website.

### COVID-19 Context

The COVID-19 pandemic led to a global surge in information seeking online in relation to the spread of the virus, best means of protection, access to health care, local rules and guidance, and, subsequently, information about COVID-19 vaccines, tracing apps and COVID-19 passports [16]. While official sources moved quickly to try and fill these information gaps, social media platforms provided a space for information and misinformation to circulate widely [17]. Conspiracy theories and rumors in relation to the virus and the vaccine were prevalent online as was poor-quality information [18-20]. The unique situation increased attention on governments as a source of information however historically government and official health sources have been subject to mistrust and their health messages resisted especially concerning vaccinations for example in the case of the Measles Mumps Rubella vaccination and the H1N1 (swine flu) vaccination program [21,22]. In these cases, trust in nonofficial information sources and the media is often higher.

### United Kingdom Context

In response to the global pandemic, the UK prime minister announced a national lockdown on March 23rd, 2023 [23]. Daily press briefings followed, led by politicians and National Health Service (NHS) leaders providing coordinated information on COVID-19 legislation and guidance, health advice, and subsequently the vaccine rollout.

Survey data indicates there was a slight increase in political trust in the United Kingdom as the lockdown commenced [24] and most people supported the government enforcement of behavior in the early months [5] with positive views on government decision-making related to response transparency. Although people looked to government and health leaders for information and guidance these officials were not immune from criticism. Politicians and advisors often found themselves at the center of news stories that challenged perceptions of trust [24], and of privacy and security, for example in relation to the rollout of contact tracing apps [25] and COVID-19 passports. Low trust in scientists and medics was also associated with COVID-19 vaccine hesitancy [26].

The sudden onset of COVID-19 and its impact not just on UK citizens but worldwide highlighted the public’s need for information. Understanding how individuals sought information from digital sources and whether they trusted this information is the focus of this study. Note that this distinct aim is different from many of the studies of information-seeking behavior during the pandemic that were more focused on the motives that drive online interrogation. Typically, these searches adopted the Risk Information Seeking and Processing model [27] which sees risk information seeking as driven by factors such as information insufficiency, subjective norms, and relevant channel beliefs. Although the Risk Information Seeking and Processing model has been used effectively to model information-seeking behaviors in relation to COVID-19 [28,29] it says relatively little about the extent to which people decide whether to trust the information they are exposed to.

Other studies have examined overall levels of trust in traditional information sources concerning COVID-19 by comparing television, radio, and newspapers with websites [30] but to our knowledge, this is the first study that examines trust and the antecedents of trust in different digital resources in relation to COVID-19. Focusing on the antecedents of trust at this time alongside individuals’ behavioral and attitudinal responses to the information they found is key for our future understanding of trusted health communication during health emergencies.

### Rationale for This Study

The revised model of trust in eHealth [15] indicates a number of antecedents for trust in online health information and advice and for intention to act on that advice. This study builds upon that work by asking whether existing trust models are a good fit for COVID-19 information-seeking online. The uncertainty provided by the COVID-19 pandemic provides a unique opportunity to examine how people search for, evaluate, and make trust decisions about health information and advice.

The COVID-19 pandemic provides an opportunity to examine in more depth the type of health information seeking that has been taking place. As described previously, people’s information needs vary including information on symptoms and symptom management, self-isolation, and vaccination. Vaccination in particular presents a unique opportunity to explore health information seeking within the context of heightened uncertainty and self-reported behavioral outcomes.

It may be that the global nature of the pandemic and people’s desire for information exchange fueled social media sources of health information and increased visibility of patient experiences. On the other hand, information corroboration is effortful, and in times of heightened stress and uncertainty, it may not be appropriate or lead to better coping outcomes. Relying on a single source of information may be more straightforward but trust in government or health professionals may impact trust perceptions around such information sources.

Therefore, the study has three aims: (1) to examine whether an existing trust model is a good fit for COVID-19–related information seeking online, (2) to examine differences in affective responses to digital resources about COVID-19 vaccination versus general information about COVID-19, and (3) to examine whether searching had a self-reported impact on vaccination decisions or attitude toward COVID-19 passports.

## Methods

### Design

A cross-sectional survey was conducted in January 2022. At this time in the United Kingdom, the Omicron variant wave had just peaked, mask use was still advised but no longer compulsory in indoor settings, and self-isolation after a positive test result was still a legal requirement. We collected quantitative data from eHealth users regarding their use of health websites in relation to COVID-19. We used Prolific to recruit a representative UK sample.

### Participants

A total of 600 people completed the survey. In total, 525 participants indicated they had looked for COVID-19 information online. Of these 85.3% (448/525) had looked for more general information and advice about COVID-19 while 14.7% (77/525) had looked for information specifically on the vaccine. Full details of participant demographics can be found in Table 1.

Participants were asked whether they had gone online to look for health advice and information about COVID-19. Those answering “yes” were asked to indicate whether they had been searching for general health advice about COVID-19 or whether they had been searching for health advice about COVID-19 vaccinations. Participants then completed a series of questions relating to the last time they searched for health advice about COVID-19 online. Specifically, they were asked to “think about any one digital source that you visited during that search” and to answer the remaining questions with respect to that source. They answered questions relating to the impact of health advice on their coping perceptions and intention to act on the advice, the degree to which they trusted the information and the digital source, their attitude toward COVID passports, for example, the NHS app that shows proof of vaccination and demographic information.

**Table 1:** Participant demographics (of those who reported looking for COVID-19 information, N=525). All participants were from the United Kingdom.

Characteristics			Values, n (%)
**Age group (years)**			
	18-25	54 (10.3)	
	26-34	85 (16.2)	
	35-54	197 (37.5)	
	55-64	123 (23.4)	
	65 years or older	66 (12.6)	
**Sex**			
	Male	249 (47.4)	
	Female	273 (52)	
	Transgender	2 (0.4)	
	Other	1 (0.2)	
**Ethnicity**			
	Caucasian	430 (81.9)	
	Latino or Hispanic	3 (0.6)	
	Middle Eastern	5 (1)	
	African	11 (2.1)	
	Caribbean	10 (1.9)	
	South Asian	31 (5.9)	
	East Asian	11 (2.1)	
	Mixed	12 (2.3)	
	Other	7 (1.3)	
	Prefer not to say	5 (1)	
**Education level**			
	Less than secondary school	2 (0.4)	
	Secondary school	68 (13)	
	Further education (eg, college, A-level)	177 (33.7)	
	Bachelor’s degree	194 (37)	
	Postgraduate degree (eg, MSc, PhD, MD)	82 (15.6)	
	Prefer not to say	2 (0.4)	
**Employment**			
	Full time	254 (48.4)	
	Part time	87 (16.4)	
	Retired	85 (16.2)	
	Unemployed	60 (11.4)	
	Student	29 (5.5)	
	Prefer not to say	10 (1.9)	
**Relationship status**			
	Single	143 (27.2)	
	Married or civil partnership or cohabiting	333 (63.4)	
	Divorced	30 (5.6)	
	Widowed	10 (1.9)	
	Prefer not to say	9 (1.7)	

### Measures

Unless stated otherwise, participants answered the following measures on a 5-point Likert scale (1=strongly disagree to 5=strongly agree).

### General Web Trust Questionnaire

The general web trust questionnaire contained 36 items from the study by Sillence et al [15] alongside measures of coping, information corroboration, and affective responses also taken from Sillence et al [15]. Specifically, coping was measured by asking participants to respond to the following stem and variables “After I read the information about COVID-19 I felt…” (1) in control and (2) optimistic using a 5-point scale with the labels: 1=less, 2=slightly less, 3=no different, 4=slightly more, and 5=more (Cronbach α=.84.). Additional affective responses, worried, reassured, at risk, confused and anxious were measured using the same format.

Information corroboration with other sources of information was measured with the following 4 items: (1) “I checked other websites,” (2) “I checked other sources,” (3) I found the advice consistent across other websites or apps, and (4) I found the advice consistent across other sources (Cronbach α=.87).

Impact on vaccination decision was measured using a single item developed for this study: “To what extent did the information and advice you read online impact your decision regarding COVID vaccinations?” Responses were given on a 5-point scale from “1=It did not influence at all” to “5=It influenced to a very large degree.”

Attitude toward COVID-19 passports was measured using a single item developed for this study, that is, “I think COVID passports are a good idea” (1=strongly disagree to 5=strongly agree).

### Outcome Measures

Trust was measured following Sillence et al [15], using the mean response to the following 2 items: (1) “I trusted the site” and (2) “I felt I could trust the information on the site” (Cronbach α=.95). Intention to act was an outcome measure, assessed with 1 item “I intended to act upon the advice.” This item was taken from Sillence et al [15].

### Ethical Considerations

The study received full ethical approval from Northumbria University ethics committee (REF:33639). The survey was hosted on Qualtrics and all data was anonymized. The first page provided participants with information detailing the aim, length, data storage, contact details, and withdrawal process of the study. They were then asked to provide informed consent. Participants received £1.25 (€1.49; US $1.66) for taking part in the study and the average completion time was around 7 minutes.

## Results

### Overview

We first explored the general web trust questionnaire by performing principal component analysis (PCA). We then explored the relationship between the factor structure and outcomes by testing its fit to the sampled data using structural equation modeling (SEM).

### Properties of the General Web Trust Questionnaire

The 36 items of the scale were entered into the PCA. All items loaded onto the extracted components but any items with factor loadings lower than 0.30 were suppressed (Table 2). The analysis indicated that 5 components possessed eigenvalues greater than 1 and together explained 68.7% of the variance in keeping with accepted conventions for successful PCA [31]. The Familiarity factor is the weakest of those extracted although it does meet the minimum threshold of comprising three items [32].

**Table 2:** Factor loadings for each item (factor loadings lower than .30 are suppressed).

Item	Rotation factor loadings				
	Personal experience (PEx)	Credibility and impartiality	Usability	Privacy	Familiarity
The language made it easy to understand	—^a^	—	.69	—	—
It helped me understand the issue better	—	—	.70	—	—
It was easy to use	—	—	.77	—	—
It told me most of what I needed to know	—	—	.59	—	—
The layout was consistent with other digital sources	—	—	.61	—	—
The advice appeared to be prepared by an expert	—	.69	—	—	—
The advice seemed to be offered in my best interests	—	.73	—	—	—
The advice came from a knowledgeable source	—	.73	—	—	—
The advice seemed credible	—	.80	—	—	—
It was owned by a well-known organization	—	—	—	—	.73
It featured familiar logos	—	—	—	—	.78
It had a professional design	—	—	—	—	.64
It had an attractive design	—	—	.47	—	—
It provided reassurances about my privacy	—	—	—	.66	—
It gave the option to post anonymously	—	—	—	.45	—
It gave reassurances about how they used your information	—	—	—	.78	—
It had a privacy policy	—	—	—	.82	—
It explained their use of cookies	—	—	—	.75	—
It contained accounts of other people’s experiences	.87	—	—	—	—
There was a chance to share my experiences	.90	—	—	—	—
There were opportunities to interact with other people on the digital source	.87	—	—	—	—
I saw a wide range of experiences rather different to mine	.88	—	—	—	—
It offered powerful accounts of health experiences	.85	—	—	—	—
It felt like the advice was tailored to me personally	.62	—	—	—	—
I was offered the chance to see experiences from people just like me	.91	—	—	—	—
It contained contributions from likeminded people	.92	—	—	—	—
I was able to contribute to content on the digital source	.88	—	—	—	—
The personal accounts on the digital source were written by people similar to me	.91	—	—	—	—
I found personal accounts that reflected my own experience	.92	—	—	—	—
I found personal accounts that were relevant to my condition	.93	—	—	—	—
There were opportunities to gather information from the personal accounts on the digital source	.91	—	—	—	—
The personal accounts contained advice for readers	.91	—	—	—	—
The personal accounts provided social or emotional support	.89	—	—	—	—
The advice appeared to be impartial and independent	—	.78	—	—	—
The advice seemed objective (ie, no hidden agenda)	—	.81	—	—	—
It was free from advertisements		.54	—	—	—
Eigenvalues	11.8	4.7	3.2	3.0	2.1
Variance explained (%)	32.7	13.1	8.9	8.2	5.8

^a^Not available.

### Exploring the Relationship Between the Trust Questionnaire and Self-Reported Behavioral Outcomes

The data were further analyzed using SEM performed in analysis of moment structures using the maximum likelihood estimation method on the item covariance matrix. The specified model was based on Sillence et al [15] and modified to incorporate the new 5-factor structure. The goodness of fit indices supports the specified model. The chi-square value indicated poor fit (*χ*^2^_773_=1265.5; *P*<.001). However, this test is considered too sensitive for samples over 200 and here the sample size is 448. The Cmin/*df* value of 1.64 indicates a good fit. The goodness of fit and adjusted goodness of fit values of .89 and .86 respectively indicate adequate fit [33]. The comparative fit index value of .97 indicates good fit [34], as does the root mean square of approximation value of .04, 90% CI .034-.041 [35].

**Figure 1:**
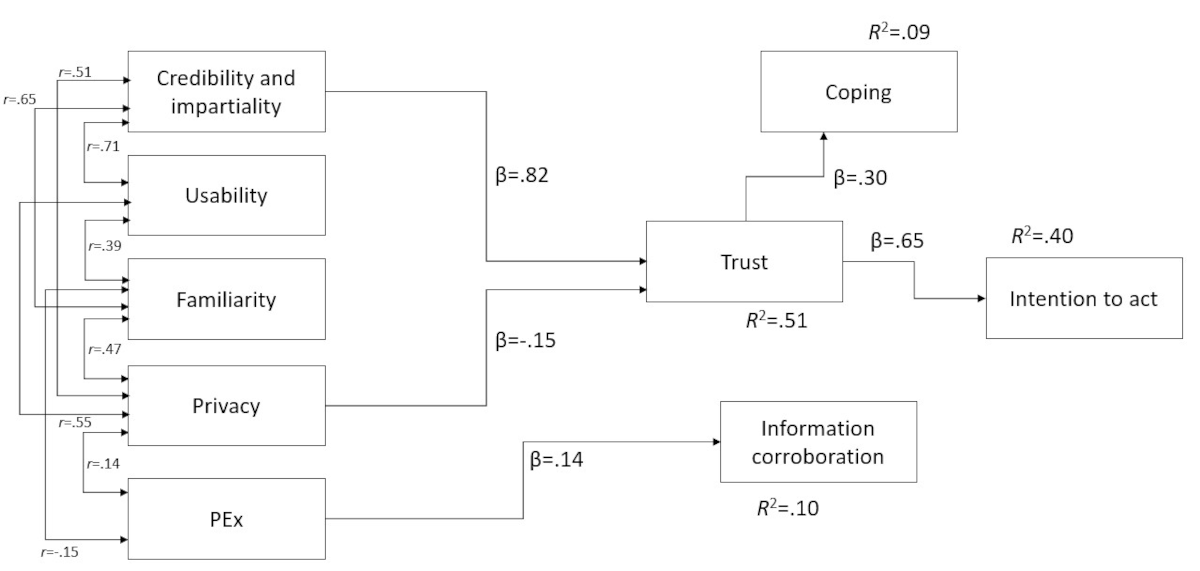
The trust model with significant standardized path coefficients.

The model accounted for 64.7% of the variance in trust, 8.7% in coping, 9.7% in information corroboration, and 40.3% in intention to act. All beta path coefficients including those in Figure 1 and those that were not significant were inspected in evaluating the predictive power of the model and are presented for completeness in Table 3.

**Table 3:** The unstandardized path weights and critical ratio (ie, *z* score) values for the main effects of the hypothesized full model.

Parameter		Unstandardized path coefficient	Critical ratio	*P* value
Credibility and impartiality				
	Trust	.93	9.79	<.001
	Information corroboration	.17	1.07	.29
Usability				
	Trust	–.05	–.36	.72
	Information corroboration	.39	1.56	.12
Familiarity				
	Trust	–.04	–.64	.52
	Information corroboration	.12	.98	.33
Privacy				
	Trust	–.19	–2.43	.02
	Information corroboration	.06	.41	.68
Personal experience				
	Trust	–.001	–.03	.98
	Information corroboration	.09	2.78	.01
Trust				
	Coping	.27	4.89	<.001
	Intention to act	.80	15.23	<.001
Coping–intention to act		–.04	–.67	.50
Information corroboration				
	Trust	.001	.03	.98
	Intention to act	–.02	–.61	.54

Only Credibility and Impartiality were found to possess a significant positive path to Trust. Privacy had a weaker yet significant negative path, meaning privacy assurances were associated with lower trust. Familiarity, usability, and personal experience (PEx) were not significantly predictive of Trust. Only Trust was found to significantly predict the intention to act on the advice. In addition, Trust significantly predicted Coping, suggesting that trustworthy websites heighten individuals’ coping perceptions, making them feel more in control and optimistic. PEx significantly predicts Information Corroboration, suggesting that people are exploring a little further than the original digital source; however, this corroboration process does not appear to be affecting their level of trust or intention to act.

### Comparison of Two Populations

Although the relatively small sample size for the vaccine information group meant that a comparable SEM model could not be reliably tested a series of independent samples *t* tests were used to compare the two groups on the key variables of interest (Tables 4 and 5).

**Table 4:** Mean (SD) values for key outcome variables.

Group	Trust	Intention to act	Corroboration	Impact on the decision regarding vaccination	Attitude toward COVID-19 passports
Searching for information on vaccinations (N=77)	4.22 (.91)	4.10 (1.05)	3.49 (1.24)	2.90 (1.21)	3.38 (1.51)
Searching for information on COVID-19 (N=448)	4.33 (.74)	4.13 (.89)	3.49 (1.06)	2.74 (1.39)	3.51 (1.36)

**Table 5:** Mean (SD) values for “after I read the information” variables.

Group	Worried	Reassured	At risk	Confused	Anxious	Optimistic	In control
Searching for information on vaccinations (N=77)	2.27 (1.11)	3.84 (.95)	2.40 (.98)	2.14 (1.13)	2.42 (1.20)	3.66 (1.11)	3.57 (1.13)
Searching for information on COVID-19 (N=448)	2.48 (.88)	3.68 (.77)	2.84 (.88)	2.15 (.98)	2.76 (.97)	3.27 (.81)	3.42 (.85)

### Independent Sample *t* tests

There was no significant difference between groups for trust (*t*_523_=–1.169; *P*=.24; Cohen *d*=–.14, 95% CI –.386 to .098), intention to act (*t*_523_=–.187; *P*=.85; Cohen *d*=–.02, 95% CI –.265 to .219), corroboration (*t*_523_=–.038; *P*=.97; Cohen *d*=–.01, 95% CI –.247 to .237), impact on decision regarding vaccination (*t*_523_=.934; *P*=.35; Cohen *d*=.115, 95% CI –.127 to .357), or COVID-19 passports (*t*_523_=–.773; *P*=.44; Cohen *d*=–.095, 95% CI –.337 to .146).

Those searching for information on vaccinations (mean 2.40) felt significantly less at risk than those searching for general information on COVID-19 (mean 2.84; *t*_523_=3.988; *P*<.001; Cohen *d*=–49, 95% CI –.735 to –.2348) and felt significantly less anxiou*s* (mean 2.42) than those searching for general information on COVID-19 (mean 2.76; *t*_523_=–2.758; *P*=.003; Cohen *d*=–.34, 95% CI –.583 to –.097). Those searching for information on vaccinations (mean=3.66) felt significantly more optimistic than those searching for general information on COVID-19 (mean=3.27; *t*_523_=3.760; *P*<.001; Cohen *d*=.464, 95% CI .220-.707).

There was no significant difference for the variable “In Control” (*t*_523_=1.335; *P*=.18; Cohen *d*=–.165, 95% CI –.077 to .407) or for “Confused” (*t*_523_=–.054; *P*=.96; Cohen *d*=–.007, 95% CI –.248 to .235). Finally, the variables “Worried” and “Reassured” approached but did not reach statistical significance (*t*_523_=–1.813; *P*=.07; Cohen *d*=–.224, 95% CI –.466 to .019 and *t*_523_=1.712; *P*=.09; Cohen *d*=.211, 95% CI –.031 to .453, respectively).

### Digital Sources of Information

Table 6 shows the digital sources used. The majority of participants used either the NHS health care sources or the governmental sources for both general information and vaccine-specific information.

Digital sources were categorized as: (1) Governmental sources: official UK government website (Gov.uk), World Health Organization, Office of National Statistics, and Centre for Disease Control. (2) NHS health care sources: any page hosted on the NHS website (nhs.uk). (3) Other health care sources: any non-NHS health care website. This included The Mayo Clinic, WedMD, patient.co.uk, and the Health Check podcast. (4) News websites: any of the mainstream news providers, the majority of those reported were the BBC. (5) Search engines: where participants did not go to one source but reported explicitly using search engines, such as Google, to intentionally search for COVID-19–related information, rather than, for example, visiting a particular source (perhaps a source perceived as authoritative or trusted), such as the NHS, government, or BBC websites, and browsing the content from there. (6) Scientific journal: any peer-reviewed journal publishing academic research. (7) Specific health condition websites: any website dedicated to a specified health condition rather than a general health website, including asthma.org and Crohn’s & Colitis UK. (8) Social media and forums: any online forum or social networking platform defined as user-driven and facilitating sharing of content, dialogue creation, and communication by and between users (in keeping with Kapoor et al, 2018 [36]). (9) Other: all instances where resources were not explicitly specified or where participants reported visiting multiple sources. All other resources are named individually in Table 6.

**Table 6:** Digital sources used.

Source	General information (N=448), n (%)	Vaccine specific information (N=77), n (%)
National Health Service health care sources	262 (58.48)	39 (50.65)
Governmental sources	64 (14.30)	11 (14.29)
Multiple resources or unspecific	37 (8.30)	13 (16.88)
News websites	30 (6.70)	3 (3.90)
Other health care sources	6 (1.34)	1 (1.30)
Social media and forums	20 (4.46)	2 (2.60)
Search engines	19 (4.24)	7 (9.09)
Zoe COVID-19 study	6 (1.34)	0 (0)
Scientific journals	1 (0.22)	0 (0)
Specific health condition websites	2 (0.45)	0 (0)
Wikipedia	1 (0.22)	0 (0)
TripAdvisor	0 (0)	1 (1.30)

## Discussion

### Principal Results

Trust continues to significantly influence self-reported intention to act on health information. In terms of trust predictors, only credibility and impartiality have a significant, direct, and positive relationship with trust. Privacy has a significant negative relationship with trust. Other predictors (familiarity, usability, and PEx) may be indirect and mediated through other trust variables. The variable PEx had a significant direct effect on information corroboration and trust was found to significantly relate to coping perceptions. The findings suggest a number of important discussion points.

First, the Sillence et al [15] trust model provides a reasonable fit for COVID-19–related health information online. Trust continues to predict intention and the credibility and impartiality of the digital source remains the strongest predictor of trust in digital health sources. However, compared to the 2019 model, the picture here is of a simpler trust process in which the credibility and impartiality factor does the “heavy lifting” in relation to trust compared to the other variables. Another key difference is the lack of a relationship between corroboration and trust. In earlier models, health information seekers looked to verify the information they found online by cross-checking with other digital and nondigital sources. Here we see only a direct relationship between the credibility and impartiality of the website and trust. One reason for this, given the predominance of the NHS as the most popular site for information and advice, is that our health information seekers are simply taking the website at face value providing it appears sufficiently credible and impartial. However, it is interesting that in an American sample, information seekers relied heavily upon often unreliable social media sources for information and advice, yet still engaged in relatively low levels of fact-checking [37] and so we must consider the possibility that people are being bombarded with so much information in relation to the pandemic that they simply switch off.

The role of PEx within digital sources is interesting here. While PEx significantly predicts information corroboration there was no subsequent relationship with trust. In the 2019 model [15] it was suggested that patient experiences can positively influence trust but only if users first corroborate the information through other sources. In our study, we suggest that people are checking up on these patient stories and experiences simply out of interest rather than as a way of assessing the trustworthiness of the information. When faced with a high degree of uncertainty and with limited detailed information, assessments of risk may be emotion-based [38], and people may well seek out other people’s personal accounts of their COVID-19 experiences. Personal accounts are often engaging and are seen as more relatable than statistical information when it comes to decision-making [39]. While PEx is now embedded within a diverse range of digital resources, those more closely associated with personal content, for example, social media platforms or individual blogs, were generally underrepresented in the data we collected. Instead, we observed a reliance on official digital sources, in particular, the NHS website and government sources. In terms of pandemic or emergency, reliance on official sources may be more commonplace. Sillence et al [15] found that the majority of UK respondents cited the NHS website as their source of health information, and McNeill, Harris, and Briggs [40] noted that the main UK source to be retweeted during the H1N1 pandemic was NHS Choices. In this study, there was little reported use of social media, which is perhaps surprising and contrasts with other recent health pandemics in which social media use and misinformation have been prevalent [37,41,42] as well as in earlier studies examining the COVID-19 pandemic and the facilitation of conspiracy theories [43,44].

Despite generally high levels of mistrust in the government’s overall handling of the pandemic [5], UK citizens still sought information from government sites. Moreover, we see a reliance on health professionals and public health information. In a time of limited information, there may be fewer options available to information seekers and individuals may be satisfied with seeking official sources of information even if they contain basic knowledge as opposed to more detailed, specific information. This contrasts with earlier work on trust in digital health information in which personalization or tailoring is seen as important to trust. People with long-term experience of a particular health condition often become experts by experience and may seek more specific, tailored digital resources to support their health conditions. This involves making more fine-grained assessments of the personal relevance of the information before deciding to trust or act upon the advice it contains [10,45] and is especially true where the condition is rare or less well known [46]. In the case of COVID-19, a worldwide pandemic affecting all age groups, it might be that generic information applicable to all sufficed in this case. There was little sense that people were checking COVID-19 information in relation to their other, pre-existing health conditions and specific health websites may not have had that information readily available. In light of research that shows how health information overload may lead to increased anxiety [47], our participants’ reliance on relatively few, authoritative websites seems like a reasonable strategy. Too much, possibly conflicting, information about COVID-19 can leave an individual feeling overwhelmed and will ultimately lead to “information avoidance,” which is clearly a poor outcome in the face of a global pandemic.

Unlike Sillence et al’s [15] 2019 model, we note that privacy has a weak negative relationship with trust. The topic of privacy was raised repeatedly in relation to the discussion of contact tracing apps and COVID-19 passports and so while not directly related to the digital source being used it may be that being asked to think about the privacy features of sources stimulates a wider consideration of privacy and mistrust. Rather than privacy policies etc. being seen as an example of good practice, the very fact that these options were present on digital sources may have served as a reminder that data are being collected, processed, and often shared. Privacy nudges may well remind people of the need to be mindful of privacy but can also raise awareness of the data that is available for collection [48,49].

Second, trust significantly predicted coping suggesting that trustworthy websites heighten individuals’ coping perceptions, making them feel able to cope. Interestingly, Wang et al [1] did not find an association between the use of the internet as an information source on COVID-19 and self-confidence in coping with COVID-19 but did not focus on trusted websites.

Looking at the affective variables in more detail for the two groups (general information seeking and vaccination information), we see that those searching for vaccination information felt more positive—specifically, they felt less at risk, less anxious, and more optimistic after reading the information. Wang et al [1] found that vaccination information sources have different effects on students’ coping appraisal of COVID-19 with information from medical personnel leading to greater knowledge about the mechanism of vaccination and greater response efficacy of vaccination compared to information from coworkers or colleagues. In terms of coping, during the H1N1 pandemic, those people who adopted a more problem-focused coping strategy including seeking out information to help solve problems were more likely to indicate they would be vaccinated [22]. In our data, those individuals who have gone looking for information about vaccination feel better for having done so.

Zheng et al [50] noted that vaccine information seeking is related to vaccination intention and suggested that health information seeking can be viewed as a coping behavior when people do not have sufficient knowledge of a particular health topic. Although seeking vaccine-related information online was also positively related to perceived vaccine information overload [50], it may be that sticking with a single trusted source is preferable for improved coping. Finally, there were no differences in terms of trust, intention to act on information, or attitude toward COVID-19 passports between participants who were searching for general COVID-19 health information versus those who had searched for vaccination information. This is unsurprising given the similarity of digital sources used.

In summary, people searching for general COVID-19 information as well as those searching for COVID-19 vaccine-specific information sought out official sources of information online. In terms of uncertainty when faced with a global emergent health concern people place their trust in familiar websites and rely on the perceived credibility and impartiality of those digital sources.

### Limitations

It is important to note that data was purposely not collected during a period of national lockdown in the United Kingdom. The vaccination program was already well underway and COVID-19 passports were very much still on the agenda. People may have sought information from alternative digital sources had data collection taken place during a period of lockdown. Focusing on the United Kingdom made sense given the local regulations and practices in place, but it would be interesting to make comparisons with other countries going forward. The reliance on the NHS website in the United Kingdom would be interesting to compare with countries where different funding models exist for example where health insurance schemes mean there is no single free at the point of service system. Vaccine hesitancy is relatively low in the U and has declined since the start of the vaccination rollout program from 10% to 3% in September 2021 [51]. Other countries, for example, France, have much higher levels of vaccine hesitancy [52], and comparisons here in relation to trust around digital health resources would warrant further investigation. Finally, it is interesting to note that although we have used a one-shot cross-sectional methodology, we mirror findings from Zhang et al [53], who examined trust over several waves earlier in the pandemic and noted a decrease in the use of social media over time and an increase in trust in government information.

### Conclusion

In conclusion, in the context of COVID-19, “credibility and impartiality” remain a key predictor of trust in eHealth resources but in comparison with previous models of trust in online health information, checking and corroborating information did not form a significant part of trust evaluations. In times of uncertainty when faced with a global emergent health concern, people placed their trust in familiar websites and relied on the perceived credibility and impartiality of those digital sources.

## Abbreviations

NHS: National Health Service
PCA: principal component analysis
PEx: personal experience
SEM: structural equation modeling

## Data Availability

The datasets generated and analyzed during this study are available in the Open Science Framework repository [54].
